# Case Report: Breast ductal carcinoma *in situ* with microinvasion: a case tracked over 13 years

**DOI:** 10.3389/fonc.2025.1560366

**Published:** 2025-08-12

**Authors:** Sibei Ruan, Jian Zhou, Rui Tang, Tao Wang, Xia Gao, Lixian Mou, Mingxi Tang

**Affiliations:** ^1^ Department of Pathology, The Affiliated Hospital, Southwest Medical University, Luzhou, Sichuan, China; ^2^ Department of Pathology, The Yaan People’s Hospital (Yaan Hospital of West China Hospital, Sichuan University), Yaan, Sichuan, China; ^3^ Department of Nuclear Medicine, The Affiliated Hospital, Southwest Medical University, Luzhou, Sichuan, China

**Keywords:** breast cancer, microinvasion, metastasis, therapy, HER2 gene

## Abstract

**Background:**

Numerous studies have reported considerable heterogeneity in breast ductal carcinoma *in situ* with microinvasion (DCIS-MI) regarding clinical presentation, progression potential, and treatment strategies.

**Case presentation:**

In this work, we report a case of rapidly progressing multiple metastases in a patient with DCIS-MI following conventional treatment. Subsequent fluorescence *in situ* hybridization (FISH) testing revealed HER2 gene overexpression. After receiving 10 cycles of trastuzumab-targeted therapy, the patient achieved a favorable prognosis and remains stable to date.

**Conclusion:**

This case suggests that even patients with microinvasive lesions who are HER2-positive may exhibit unexpected stability under targeted therapy, warranting close surveillance in such cases.

## Introduction

Breast ductal carcinoma *in situ* with microinvasion (DCIS-MI) is classified as a T1mi tumor according to the American Joint Committee on Cancer (AJCC) Staging, 8th edition, defined by stromal invasion extending beyond the basement membrane with foci measuring ≤1 mm. Epidemiological studies have indicated that DCIS-MI occurs in approximately 5%–10% of DCIS cases (1–3). Its incidence has risen over recent decades due to widespread mammographic screening, color Doppler ultrasound, and increased clinical awareness. Numerous studies have reported considerable heterogeneity in DCIS-MI regarding clinical presentation and progression potential. Some scholars have considered DCIS-MI an early form of invasive breast carcinoma (IBC), suggesting comparable mortality and 5-year overall survival rates to IBC (4–6). However, recent studies have distinguished DCIS, DCIS-MI, and IBC (7, 8), indicating that DCIS-MI patients exhibit worse survival than DCIS patients but better outcomes than IBC patients (9). Significant controversy persists regarding the pathological characteristics, diagnosis, and treatment of DCIS-MI, necessitating further research.

In this study, we report the progression and prognosis of a DCIS-MI patient with multi-organ metastases following surgery at the Affiliated Hospital of Southwest Medical University. We also discuss current consensus and controversies in DCIS-MI management based on the latest literature. The patient provided written informed consent for the publication of this manuscript and any identifying images or data. This report details one of the longest prospective follow-ups of HER2+ DCIS-MI, addressing a gap in long-term outcome data.

## Case presentation

**Figure 1:**
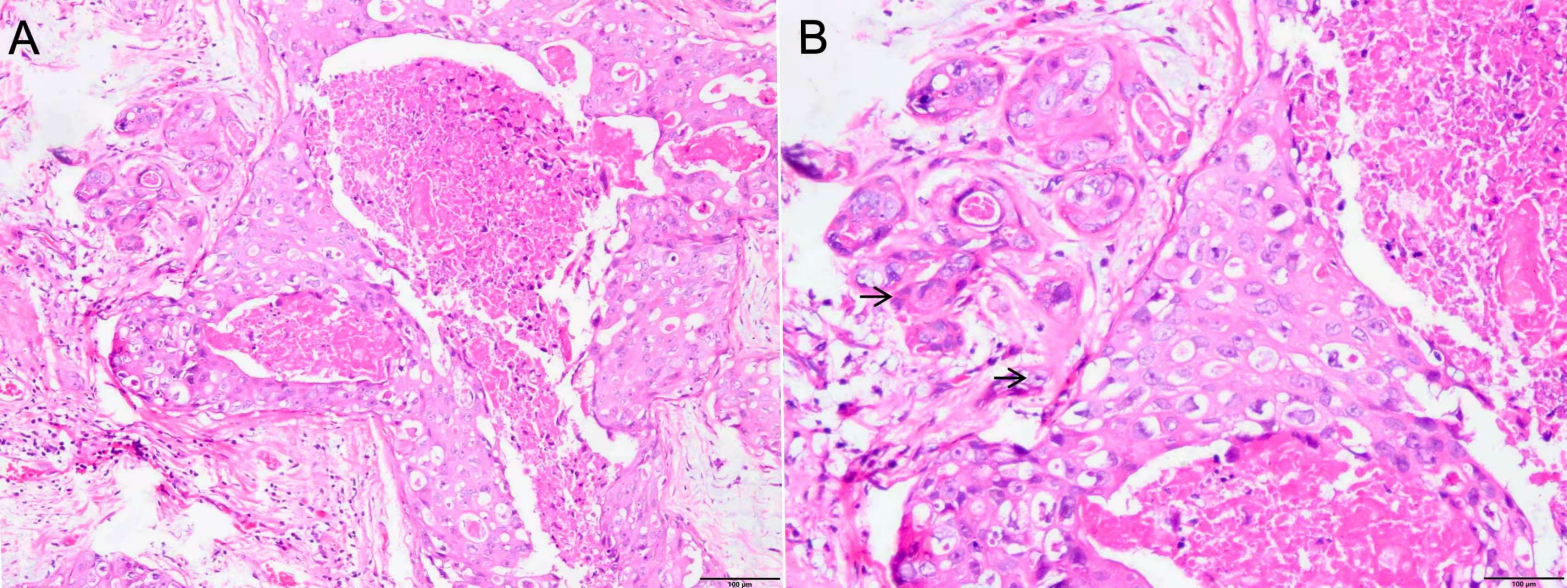
**(A)** Ductal carcinoma *in situ* of the left breast tissue with microinvasion (×100). **(B)** Ductal carcinoma *in situ* of the left breast tissue with two discrete microinvasive foci (each <1 mm) (black arrow, ×200).

**Figure 2:**
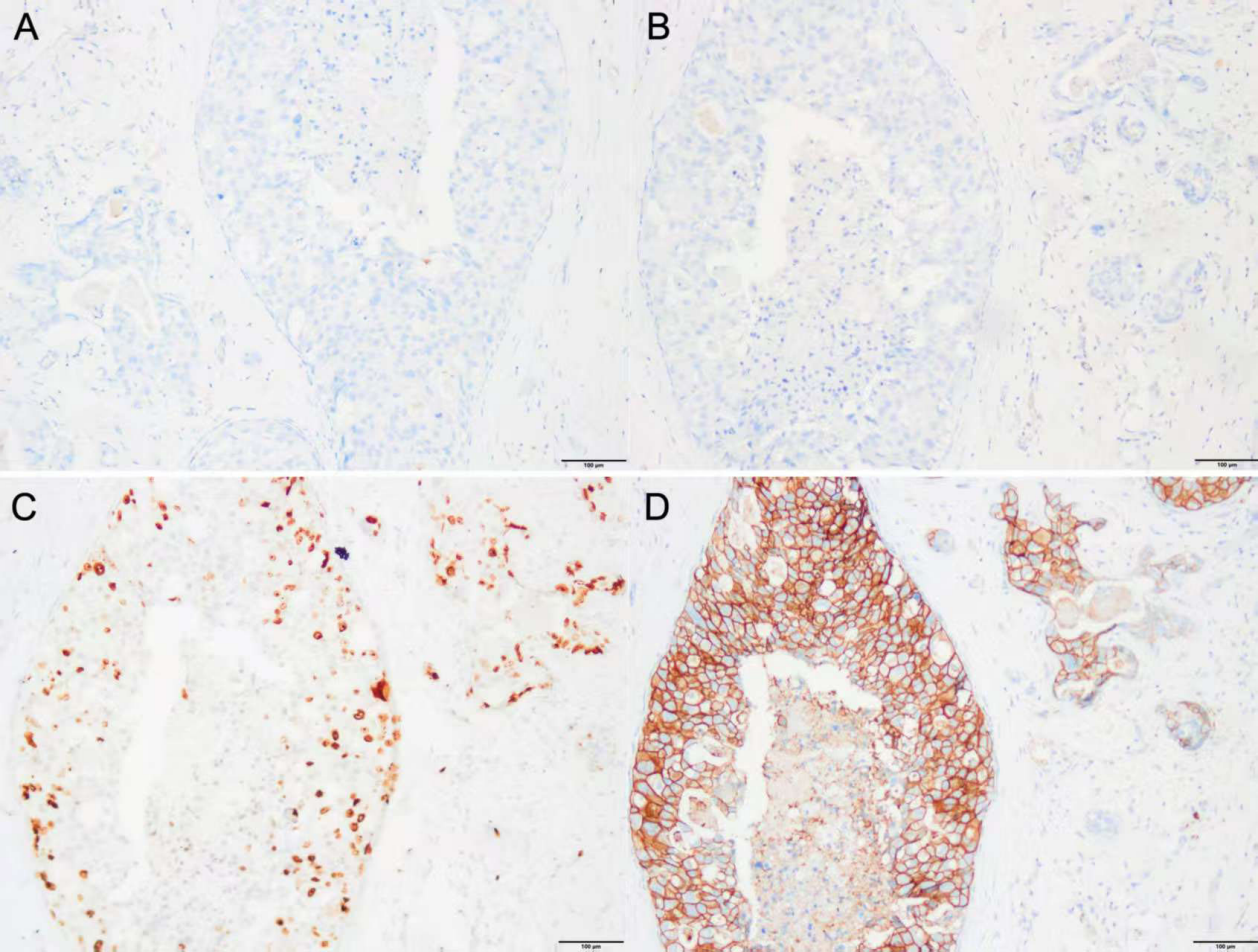
**(A–D)** IHC of the tissue of excisional biopsy of the left breast in 2012 (×200). **(A)** IHC for ER (−), **(B)** IHC for PR (−), **(C)** IHC for Ki-67 (+, 20%), and **(D)** IHC for HER2 (3+). IHC, immunohistochemistry; ER, estrogen receptor; PR, progesterone receptor; HER2, human epidermal growth factor receptor 2.

In March 2012, a 58-year-old female patient presented to the Department of Breast Surgery at our hospital with a left-sided breast mass. Physical examination revealed a 2.0-cm firm, fixed mass in the upper outer quadrant of the left breast. Mammography identified an irregular 2.0-cm mass with pleomorphic calcifications [BI-RADS (Breast Imaging Reporting and Data System) category 5]. Ultrasound demonstrated a hypoechoic lesion with angular margins. An ultrasound-guided core needle biopsy (CNB) subsequently confirmed high-grade ductal carcinoma *in situ* (DCIS) with focal microinvasion. On March 8, 2012, the patient received one course of TAC (docetaxel + doxorubicin + cyclophosphamide) induction chemotherapy. A left modified radical mastectomy was performed on April 3, 2012. Postoperative pathological examination identified two discrete microinvasive foci (each <1 mm) within a 1.5 cm area of DCIS. The radiological size (2.0 cm on mammography) correlated with the pathological DCIS extent (1.5 cm) (**Figures 1A, B**). Examination of 19 left axillary lymph nodes showed no metastases. The entire lesion was serially sectioned at 3-mm intervals. All suspicious foci were embedded in paraffin, and ≥15 additional deeper sections per block were evaluated to exclude occult IBC. Exhaustive sectioning of the tumor bed and surrounding parenchyma revealed no further invasive foci. Immunohistochemistry of the microinvasive areas showed ER (estrogen receptor, −), PR(progesterone receptor, −), Ki-67 (+20%), and HER2 (3+) (**Figures 2A–D**). Immunohistochemical staining for p63 and SMMHC (smooth muscle myosin heavy chain) revealed a continuous layer of positive cells in DCIS lesions, while no positive staining was observed around the two discrete microinvasive lesions, confirming that these nests represent invasive breast cancer foci (micrometastasis). Following surgery, the patient completed five courses of TAC chemotherapy and 4 weeks of conventional radiotherapy. Post-chemotherapy re-examination revealed no breast mass recurrence [the patient declined HER2 fluorescence *in situ* hybridization (FISH) testing].

**Figure 3:**
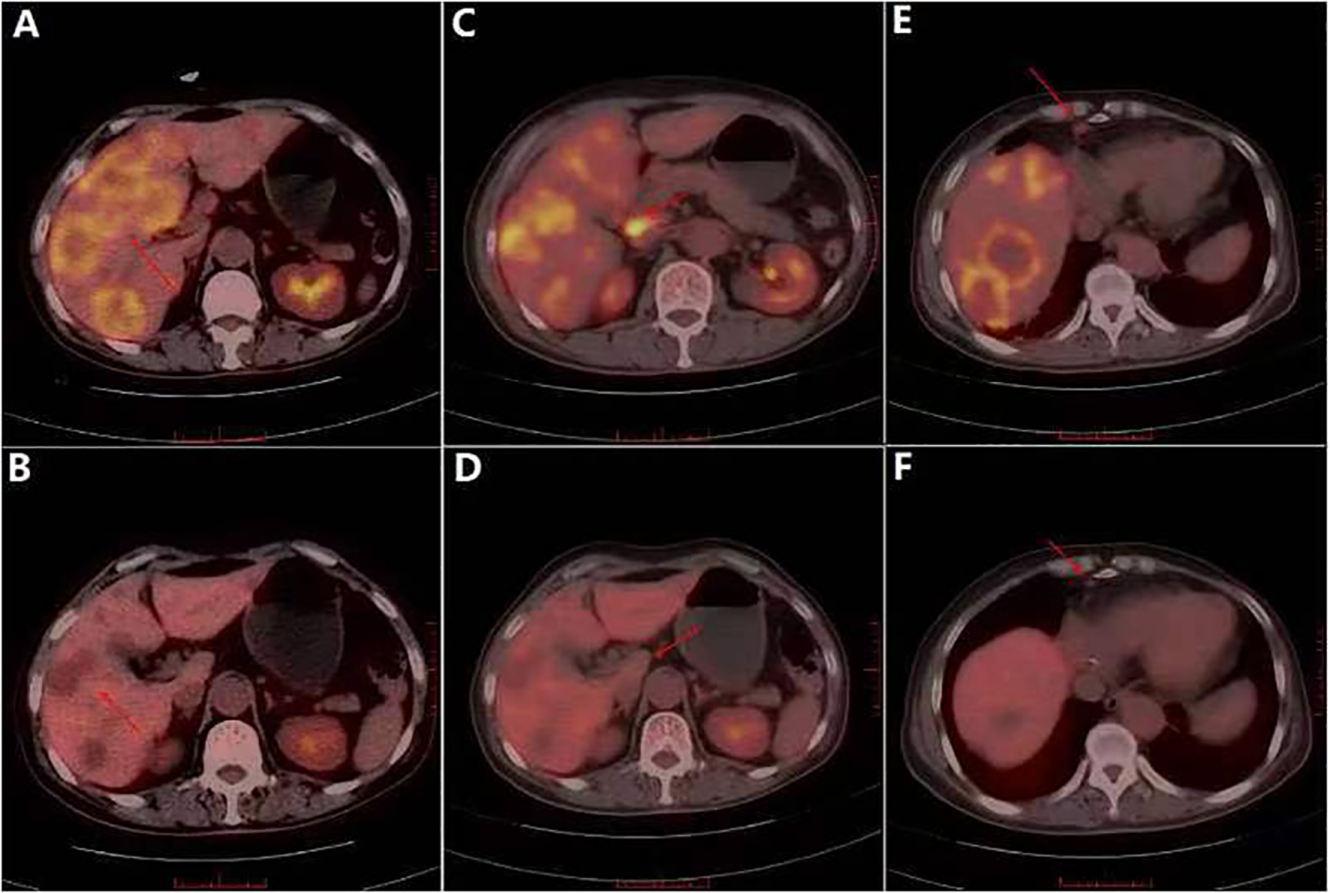
In August 2013, PET/CT showed liver and multiple lymph node metastases. **(A)** PET/CT showed multiple patchy and flaky low-density shadows of different sizes in the liver. **(B)** PET/CT showed the disappearance of patchy low-density shadows in the liver. **(C)** Multiple lymph node enlargement and increased glucose metabolism in right septal angle. **(D)** No swelling or increased glucose metabolism was observed in the right septal horn lymph node. **(E)** Local glucose metabolism of the sacrum was increased. **(F)** Local glucose metabolism of the sacrum was reduced.

**Figure 4:**
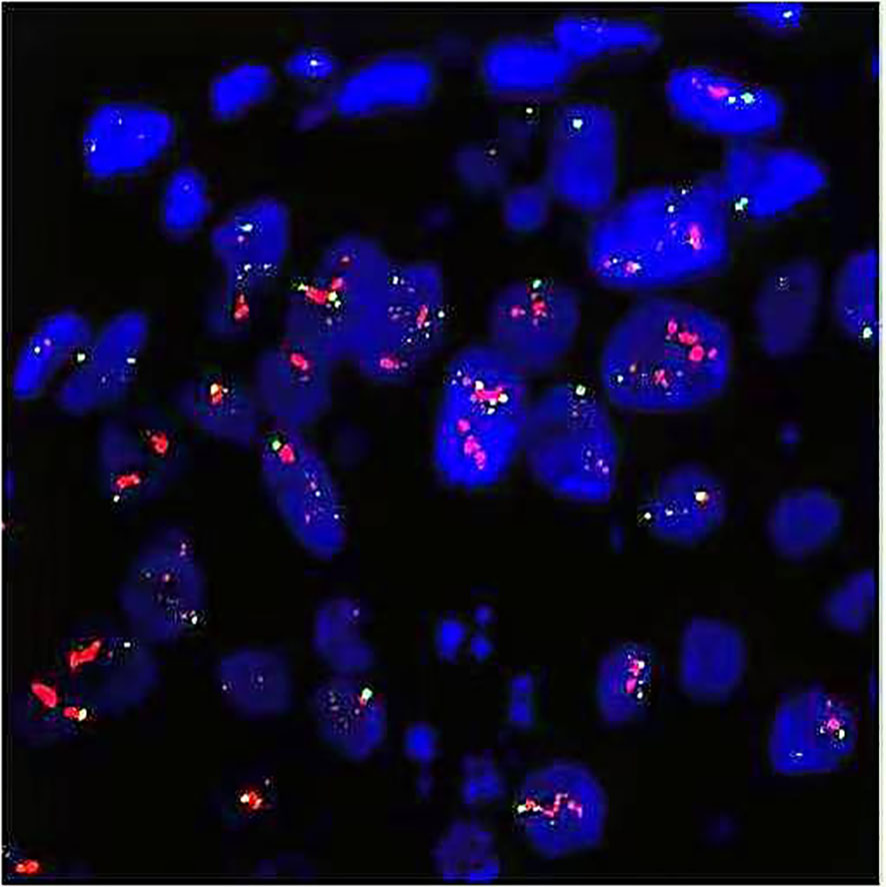
FISH test indicated HER2 overexpression (cluster amplification of HER2 signals in microinvasive carcinoma cells). Red for HER2 gene and green for CEP17. FISH, fluorescence *in situ* hybridization; CEP, centromere encoded probe.

On August 14, 2013, the patient presented to the Department of Hepatobiliary Surgery at our hospital with upper abdominal pain. Ultrasound revealed substantial intrahepatic space-occupying lesions and multiple enlarged retroperitoneal lymph nodes. PET/CT showed (**Figures 3A, C, E**) the following: 1) multiple variably sized patchy and flaky low-density lesions in the liver with heterogeneously increased glucose metabolism, suggestive of breast cancer metastasis given the patient’s history; 2) multiple enlarged lymph nodes with increased glucose metabolism in the right cardiophrenic angle, porta hepatis, and retroperitoneal space, consistent with lymph node metastases from breast cancer; and 3) focally increased glucose metabolism in the sacrum, raising suspicion of breast cancer metastasis. On August 16, 2013, with the patient’s consent, the FISH test confirmed HER2 overexpression (cluster amplification of HER signals in microinvasive cancer cells) in both the pretreatment CNB and surgical specimen, per the American Society of Clinical Oncology / College of American Pathologists (ASCO / CAP) 2008 (**Figure 4**). Liver biopsy was deferred per institutional guidelines due to the patient’s rapid clinical deterioration and strong clinical/radiological evidence supporting a primary breast origin. Based on the National Comprehensive Cancer Network (NCCN) guidelines for HER2+ disease, treatment with trastuzumab (6 mg/kg every 3 weeks) plus capecitabine (1,250 mg/m^2^ twice daily on days 1–14 of a 21-day cycle) was initiated. The patient received eight courses of this regimen from August 17, 2013, to February 6, 2014. PET/CT re-evaluation after three courses showed a significant reduction in the size and metabolic activity of the intrahepatic lesions compared to those in August 2013. Post-treatment PET/CT on March 15, 2014 (**Figures 3B, D, F**) revealed the following: 1) hepatic cyst and calcification in the left lateral liver lobe, 2) partial mediastinal lymph node calcification, and 3) normal bone scan and CT findings. From March 14, 2014, to December 9, 2014, she received an additional 10 courses of trastuzumab plus capecitabine. Maintenance therapy with oral capecitabine (2.5 g twice daily) was followed from January 14, 2015, to January 12, 2016 (10 courses). Quarterly PET/CT surveillance during this period showed no abnormalities. In February 2016, the patient developed grade 2 hand–foot syndrome. Capecitabine dosing was reduced to 2.0 g twice daily, administered from February 5, 2016, to March 15, 2017 (16 courses). A PET/CT scan in March 2017 showed no new lesions or metastases. The patient continued single-agent capecitabine chemotherapy from April 2017 to January 2021. Semiannual PET/CT scans during this period showed no significant changes compared to the 2017 baseline. Since February 2021, all chemotherapy treatments have been discontinued. The patient has been undergoing annual CT surveillance and physical assessment, with no abnormalities detected to date. This case was identified through retrospective review of our institutional breast cancer registry (IRB#12732). Data collection adhered to STROBE (strengthening the reporting of observational studies in epidemiology) guidelines for case reports.

## Discussion

The patient was diagnosed with DCIS-MI based on biopsy and subsequent surgical excision. Histopathological examination of H&E-stained sections revealed two discrete microinvasive foci, each measuring <1 mm in the greatest dimension. Negative immunohistochemical staining for p63 and SMMHC confirmed that these foci represented invasive breast cancer (micrometastasis). However, due to prolonged storage and significant fading of the original slides, images from that time could not be retrieved. Additionally, re-staining the corresponding paraffin blocks failed to produce images of diagnostic quality. The absence of a myoepithelial marker immunohistochemistry image represents a limitation in this case. Notably, the patient developed widespread systemic metastases within a short timeframe. This case presents a paradoxical clinical course: while the majority of lesions represented DCIS with only minimal invasive components (<1 mm), the disease exhibited aggressive metastatic behavior atypical of pure DCIS. We elucidate the clinical significance of this case through a comparative analysis of the patient’s age, tumor dimensions, histopathological characteristics, biomarker profile, axillary lymph node status, and targeted therapy regimen.

First, regarding age, studies have indicated that DCIS-MI patients are typically older than those with pure DCIS (10, 11). In this case, the patient was 58 years old at diagnosis (postmenopausal), consistent with literature suggesting age >50 years as a potential independent risk factor for microinvasion (12, 13). We thus speculate that prolonged clonal evolution may drive DCIS microinvasion: extended lesion duration could permit the emergence of aggressive subclones through stochastic genetic alterations, increasing invasive potential. Second, concerning tumor size, Jia et al. (14) reported that 61.6% of DCIS-MI lesions exceed 3 cm in diameter, while Mori et al. (10) documented a median tumor diameter of 3.5 cm. These data suggest that larger tumors (particularly >2 cm) correlate with microinvasion risk. Notably, this patient’s tumor measured only 1.5 cm yet developed multiple metastases. We therefore postulate that while DCIS lesion size contributes to invasive potential, the presence of microinvasion itself constitutes a critical risk factor for disease progression.

Histopathologically, DCIS-MI typically presents as higher-grade lesions across histological subtypes compared to pure DCIS (15–18). Studies have indicated that 85.0% of DCIS-MI cases exhibit high-grade lesions, whereas only 63.5% of pure DCIS cases show high-grade histology. This suggests that high-grade DCIS may be a necessary precursor to most invasive carcinomas, with low-grade DCIS potentially progressing to high-grade disease before invasion. In the present case, the histological features align with reported high-grade DCIS (**Figure 1**). However, the clinical outcome diverged significantly from pure DCIS: the patient developed extensive systemic metastases rapidly, far exceeding the 15-year distant metastases rate of 0.9% observed in the NSABP B-17/24 cohort (19). This implies that DCIS-MI and pure DCIS may represent fundamentally distinct entities. We hypothesize that DCIS-MI exhibits inherent aggressiveness, progressing to invasive carcinoma more rapidly. This could explain the patient’s swift disease progression and early metastases. Biological markers further differentiate DCIS grades (20–23). Well-differentiated/low-grade DCIS typically expresses ER/PR (90%–100%), rarely shows HER2 amplification (~10%), and has low Ki-67 indices (5%–6%). In contrast, our patient’s immunohistochemistry revealed ER/PR negativity, HER2 amplification, and elevated Ki-67 (**Figure 2**). This HER2-enriched molecular subtype correlates with higher proliferation indices and microinvasion risk, potentially explaining early metastasis. Such aggressive DCIS-MI subtypes align with rare reports (24) and suggest that molecular biology may supersede conventional risk stratification. Collectively, the loss of ER/PR, HER2 overexpression, and elevated Ki-67 may serve as predictors of poor prognosis in DCIS-MI.

Published data have indicated metastatic axillary lymph node involvement in DCIS patients at frequencies of 0%–10% (25, 26), with an overall sentinel lymph node (SLN) positivity rate of 7.4% (27). Consequently, current guidelines from the NCCN and ASCO suggest that sentinel lymph node biopsy (SLNB) may be omitted in DCIS patients undergoing breast-conserving therapy (BCT) (28, 29). Conversely, the European Society for Medical Oncology (ESMO) recommends SLNB for high-grade DCIS with tumors exceeding 4 cm (30). In this case, the 1.5-cm lesion did not undergo SLNB, and axillary lymph node dissection revealed no metastases. However, the patient developed multi-organ metastases shortly after surgery. This implies that early sentinel lymph node metastasis cannot be excluded, underscoring SLNB’s diagnostic value. Our findings indicate that SLNB remains clinically significant for DCIS-MI patients, even when lesions are small (<4 cm).In addition, trastuzumab-targeted therapy is standard for HER2-overexpressing IBC. However, its efficacy in HER2-positive DCIS-MI remains unconfirmed by clinical trials. Studies have indicated that ~40% of DCIS cases are HER2-positive (31), yet insufficient evidence exists for trastuzumab’s ability to prevent DCIS progression to IBC; it currently serves only as a risk stratification reference. Consequently, trastuzumab is not standard care for HER2+ DCIS. The NSABP B-43 trial—the first prospective phase III study evaluating trastuzumab’s impact on local recurrence in high-risk HER2+ DCIS (32)—found no significant histological, antiproliferative, or pro-apoptotic effects from trastuzumab monotherapy. Although trastuzumab mediates NK cell-dependent ADCC (antibody-dependent cell-mediated cytotoxicity) and induces T-cell humoral immunity, no substantial toxicity was observed in DCIS patients, and the trial remains ongoing. Cobleigh et al. (33) investigated trastuzumab combined with radiotherapy for HER2+ DCIS but found that prognostic benefits lacked statistical significance. In this case, trastuzumab targeting HER2 (10 cycles) achieved complete remission after metastasis (**Figure 3**), suggesting clinical utility in metastatic DCIS-MI. However, as this represents a single case, therapeutic efficacy requires validation through larger trials. Notably, biomarker discordance between primary and metastatic sites occurs in 10%–20% of cases (34). We acknowledge this limitation and recommend metastatic lesion biopsy to reassess receptor status when feasible.

## Conclusion

In this case, multiple metastases of the patient display the malignant nature of DCIS-MI, and while DCIS lacks metastatic potential, microinvasion represents the transition to IBC. This case underscores that even minimal invasive components in DCIS-MI can drive distant metastases, warranting aggressive surveillance. For DCIS-MI patients, HER2 testing should be considered in DCIS-MI given potential therapeutic implications, although broader adoption requires cohort validation. In addition, a comprehensive evaluation is required for tumor size, ER and PR expression, lymph node metastases, etc. The treatment for DCIS-MI patients is not invariable; clinicians need to know more about the commonness and individuality of patients to manage more individualized treatment plans for different DCIS-MI patients. At the same time, a more reasonable and accurate method is needed to predict the possibility of different patients developing IBC and give different treatments to different subtypes of patients.

## Data Availability

The original contributions presented in the study are included in the article/supplementary material. Further inquiries can be directed to the corresponding author.
